# Upper Extremity Fractures in Older Medicolegal Patients: Radiologic Characteristics and Factors Associated with Permanent Functional Impairment

**DOI:** 10.3390/diagnostics16203399

**Published:** 2026-10-19

**Authors:** Adnan Çelikel, Ümit Şimşek, Kenan Karbeyaz, Alper Özkök

**Affiliations:** 1 Department of Forensic Medicine, Hamidiye Faculty of Medicine, University of Health Sciences Türkiye, Istanbul 34668, Türkiye; 2 Department of Forensic Medicine, Faculty of Medicine, Eskişehir Osmangazi University, Eskişehir 26000, Türkiye; umit.simsek@ogu.edu.tr (Ü.Ş.);; 3 Department of Forensic Medicine, Faculty of Medicine, Gazi University, Ankara 06500, Türkiye

**Keywords:** older adults, upper extremity fractures, forensic medicine, medicolegal assessment, functional impairment, fracture displacement, intra-articular fracture, radiography

## Abstract

**Background/Objectives:** Upper extremity fractures in older adults may cause lasting disability, but radiologic characteristics associated with permanent functional impairment in medicolegal populations remain unclear. We described these injuries and examined associated factors. **Methods:** Of the 94 initially identified patients, seven with unavailable follow-up examination records were excluded. This single-center retrospective cohort included 87 medicolegal patients aged ≥65 years with 104 radiographically confirmed upper extremity fractures evaluated between 2015 and 2024. The outcome was permanent functional impairment documented in a definitive medicolegal report after clinically appropriate follow-up. An exploratory, clinically selected logistic regression model included age, multiple fractures, displacement, and intra-articular extension. **Results:** Median age was 70 years, 70.1% were men, and interpersonal violence was the most frequent mechanism (51.7%). Permanent functional impairment was identified in 35 patients (40.2%; 95% CI, 30.6–50.7). Multiple upper extremity fractures (aOR, 3.79; 95% CI, 1.01–14.25), at least one displaced fracture (aOR, 4.52; 95% CI, 1.30–15.73), and at least one intra-articular fracture (aOR, 3.94; 95% CI, 1.13–13.77) were associated with impairment in the adjusted model; no statistically significant association was detected for age. **Conclusions:** Fracture burden and morphology were associated with permanent functional impairment; an age association was not detected in this relatively small, age-concentrated cohort. Standardized reporting of fracture number, displacement, and intra-articular extension may support long-term follow-up and medicolegal evaluation.

## 1. Introduction

Fractures in older adults are an important clinical and public health problem because age-related skeletal fragility and the risk of falling increase concurrently. Osteoporosis, frailty, and an increased propensity to fall contribute to fractures after low-energy trauma [1,2,3,4]. In a nationwide analysis of more than one million fractures among people aged 65 years or older in Türkiye, the wrist was the second most frequently affected region after the hip, and wrist fractures were particularly prominent in the 65–74-year age group [5]. These findings underscore the substantial contribution of upper extremity fractures to the burden of geriatric trauma.

Although upper extremity fractures are not consistently among the most immediately life-threatening injuries, pain, restricted motion, and loss of grip strength at the wrist, elbow, and shoulder girdle may compromise dressing, feeding, personal care, and the use of assistive devices. Proximal humerus and distal radius fractures are common in older adults, and functional outcomes are related not only to anatomic location but also to fracture morphology and treatment [6,7,8]. Evaluating features such as multiple fractures, displacement, comminution, and intra-articular extension may therefore be important for understanding long-term functional outcomes.

Radiography is central to fracture detection and morphologic characterization. Recent studies on radiographic fracture detection and automated classification of upper extremity fractures illustrate the diagnostic interest in standardized, reproducible interpretation of imaging data [9,10]. However, the relationship between imaging-defined fracture features and permanent functional impairment identified at the conclusion of medicolegal assessment remains insufficiently characterized, particularly in older medicolegal patients.

Older medicolegal patients may differ from the general population with osteoporotic fractures because of the context of injury and referral selection. Turkish medicolegal series include road traffic collisions, interpersonal violence, and other traumatic events in addition to falls; the distributions of injury mechanisms and fractures vary according to age, sex, and referral setting [11,12,13,14,15,16]. Severe fractures have also been reported more frequently among medicolegal patients aged over 65 years [16]. Consequently, observations from general geriatric fracture populations may not fully represent injury patterns and long-term outcomes among patients referred for medicolegal evaluation.

In Turkish forensic medicine practice, fractures are graded according to their effect on the functions of life, and persistent weakening or loss of organ or extremity function after healing is assessed separately [17,18,19]. Evidence that the treatment approach is associated with persistent sequelae in extremity fractures further supports the clinical basis of this evaluation [20]. Nevertheless, studies integrating demographic characteristics, injury mechanisms, radiologic fracture morphology, treatment, and final medicolegal functional outcomes in patients aged 65 years or older remain limited. We aimed to describe the demographic, trauma-related, orthopedic, and medicolegal characteristics of upper extremity fractures in older medicolegal patients and to examine associations between fracture features and permanent functional impairment in the final medicolegal report. We hypothesized that multiple fractures, displacement, and intra-articular extension would be associated with a higher probability of permanent functional impairment.

## 2. Materials and Methods

### 2.1. Study Design and Setting

This single-center retrospective observational cohort study was conducted in the Department of Forensic Medicine, Faculty of Medicine, Eskişehir Osmangazi University. It included medicolegal patients who were evaluated in the forensic medicine outpatient clinic between 1 January 2015 and 31 December 2024, were aged 65 years or older at the time of the event, and had a traumatic upper extremity fracture.

### 2.2. Study Population

Patients were eligible if they were aged ≥65 years at the time of the event, sustained at least one radiologically confirmed upper extremity fracture after acute trauma, and underwent medicolegal evaluation for the same event. During the study period, 94 patients meeting these initial criteria were identified. Seven were excluded because follow-up examination records were unavailable and their final functional outcomes could not be reliably ascertained. The final analytic cohort comprised 87 patients (92.6% of initially identified cases); no further sampling was performed. A minimum follow-up of 18 months was not an eligibility requirement. Definitive reports could be issued earlier following full functional recovery, whereas decisions regarding permanent functional impairment were deferred until at least 18 months after injury and until no further meaningful recovery was anticipated.

Patients evaluated outside the study period, those aged <65 years at the time of the event, those without an acute traumatic upper extremity fracture, those with a pathologic fracture or only sequelae of a previous fracture, those with insufficient core clinical or medicolegal records, and those with duplicate presentations were excluded. For patients with more than one upper extremity fracture, each patient contributed once to patient-level analyses, whereas each fracture was treated as a separate observation in descriptive fracture-level analyses.

### 2.3. Data Sources and Variables

Data were obtained from forensic medicine records, the hospital information management system, and the original post-injury radiology reports issued by radiologists. Each patient was assigned a unique study code without personal identifiers. Patient-level variables included age, sex, living arrangement, injury mechanism and subtype, trauma energy category, suspected abuse or neglect, comorbidities, associated injuries, fractures outside the upper extremity, treatment, hospital admission, length of stay, 30-day revisit, complications, and medicolegal outcomes.

Fracture-level variables included side, affected bone and anatomic segment, displacement, comminution, intra-articular extension, open or closed status, fracture-dislocation, vascular or nerve injury, and fracture-specific treatment. Injury mechanisms were classified as interpersonal violence, road traffic collision, fall, occupational accident, entrapment/crush injury, or other. Road traffic collisions and falls were further divided into predefined subcategories in the source records. Trauma energy was grouped as low-to-moderate or high according to the mechanism-based classification in the study coding form; the low and moderate categories were combined for analysis because of small cell counts.

Radiologic characteristics, including displacement, comminution, and intra-articular extension, were extracted from the original post-traumatic radiology reports prepared by radiology specialists. These characteristics were documented in the source reports for the included fracture records; thus, no missing-value assumption was used to classify an unmentioned characteristic as absent. Displacement and comminution were classified according to the reporting radiologist’s description, and intra-articular extension according to documented joint-surface involvement. The source images were not independently re-evaluated for this study. Accordingly, there was no research-specific blinded image assessment or interobserver agreement analysis. Because no uniform study-specific quantitative imaging thresholds were applied, variation among radiologists’ reporting practices remains possible.

### 2.4. Outcomes

The timing of the definitive medicolegal report depended on clinical recovery. When the initial assessment was inconclusive, repeat clinical examination was requested in the preliminary report, and follow-up attendance was facilitated through the judicial process, including reminders coordinated by law enforcement. Patients with complete functional recovery could receive a definitive report before 18 months. In contrast, suspected persistent deficits were reassessed periodically, and a determination of permanent functional weakening or loss was deferred until at least 18 months after injury, when further meaningful recovery was no longer anticipated. The final clinical medicolegal assessment was routinely performed by at least two forensic medicine specialists. If a definitive conclusion could not be reached, a third forensic medicine specialist participated. When clinically indicated, orthopedic surgery or physical medicine and rehabilitation specialists were consulted; however, the final medicolegal determination remained the responsibility of the forensic medicine specialists. The assessment was based primarily on persistent injury-related clinical deficits, particularly restricted joint range of motion and, where applicable, decreased muscle strength, contracture, motor or sensory deficits, and limitations in functional use of the upper limb. Fracture displacement, comminution, and intra-articular extension were not themselves criteria for determining permanent functional weakening or loss. Physicians undertaking the final assessment could have access to the earlier imaging reports; formal blinding was not performed.

The distinction between permanent functional weakening and permanent loss of function was based on the national medicolegal guideline for the evaluation of bodily injuries under the Turkish Penal Code [19]. According to this guideline, permanent anatomical or functional impairment involving approximately 10–50% of the affected organ or extremity is classified as permanent functional weakening, whereas impairment exceeding 50% is classified as permanent loss of function. The final classification was based on the clinical findings and medicolegal assessment of the forensic medicine specialists.

The primary outcome was permanent trauma-related impairment of sensory or organ function documented in the final medicolegal report. The categories of permanent functional weakening and permanent loss of function were combined into a binary outcome; patients without permanent functional impairment formed the reference group. Secondary outcomes were the separate distributions of functional weakening and functional loss, the anatomic region and type of impairment, hospital admission, 30-day revisit, complications, and treatment modality.

### 2.5. Statistical Analysis

The distributions of continuous variables were assessed using histograms, quantile–quantile plots, and descriptive statistics. Continuous variables are presented as medians and interquartile ranges (IQRs), and categorical variables as counts and percentages. The 95% confidence interval (CI) for the primary outcome proportion was calculated using the Wilson method. Pearson’s chi-square test was used for categorical comparisons when all expected cell counts were ≥5; otherwise, the two-sided Fisher’s exact test was applied. Continuous variables were compared using the Mann–Whitney U test.

Fracture-level observations were not treated as independent in patient-level inferential analyses. For each morphologic feature, a patient-level binary variable indicated whether at least one fracture had that feature. Given the limited number of 35 outcome events, the multivariable logistic regression model was restricted to four clinically selected variables: age per 5-year increase, multiple upper extremity fractures, at least one displaced fracture, and at least one intra-articular fracture. Automated variable selection was not used. Treatment modality, hospital admission, revisit, and complications were not entered because they could occur after injury severity and act as intermediate or outcome variables. Results are reported as adjusted odds ratios (aORs) with 95% CIs. The model was intended to describe adjusted associations, not to estimate or validate individual predictive performance. Fracture localization and other potentially relevant baseline variables were not incorporated into the original model; residual confounding cannot be excluded. No additional sensitivity analysis was performed.

No data were missing for the primary outcome or model variables; therefore, no imputation was performed, and complete-case analyses were used. Comparisons were exploratory, and no adjustment for multiple testing was applied. All tests were two-sided, with *p* < 0.05 considered statistically significant. Analyses were conducted using Python 3.12 (Python Software Foundation, Wilmington, DE, USA), pandas 2.2.3 (pandas development team), and SciPy 1.17.0 (SciPy developers).

### 2.6. Ethics

The study was conducted in accordance with the Declaration of Helsinki and was approved by the Ethics Committee of Eskişehir Osmangazi University.

## 3. Results

### 3.1. Patient Characteristics

Of the 94 initially identified patients aged ≥65 years with upper extremity fractures, seven (7.4%) were excluded because follow-up examination information was unavailable. The final analytic cohort comprised 87 patients (92.6%). The median age was 70 years (IQR, 68–74; range, 65–91), and 61 patients (70.1%) were men. Sixty-seven patients (77.0%) were aged 65–74 years, 16 (18.4%) were aged 75–84 years, and 4 (4.6%) were aged ≥85 years. Sixty-eight patients (78.2%) lived with a spouse or family, and 18 (20.7%) lived alone.

The most frequent injury mechanism was interpersonal violence (*n* = 45; 51.7%), followed by road traffic collisions (*n* = 24; 27.6%) and falls (*n* = 10; 11.5%). Twelve cases of interpersonal violence occurred in the context of domestic violence, and suspected abuse or neglect was recorded in 10 patients (11.5%). Twenty-seven patients (31.0%) had another associated injury, and 15 (17.2%) had an additional fracture outside the upper extremity.

Forty-nine patients (56.3%) received conservative treatment only, 32 (36.8%) received surgical treatment only, and 6 (6.9%) received combined treatment. Twenty-three patients (26.4%) were admitted to hospital; among admitted patients, the median length of stay was 8 days (IQR, 4–11). Fourteen patients (16.1%) revisited within 30 days, and at least one complication occurred in 15 (17.2%). The median time from the event to forensic medicine evaluation was 12 days (IQR, 5.5–46). Patient-level characteristics are presented in Table 1.

### 3.2. Fracture Characteristics

**Figure 1:**
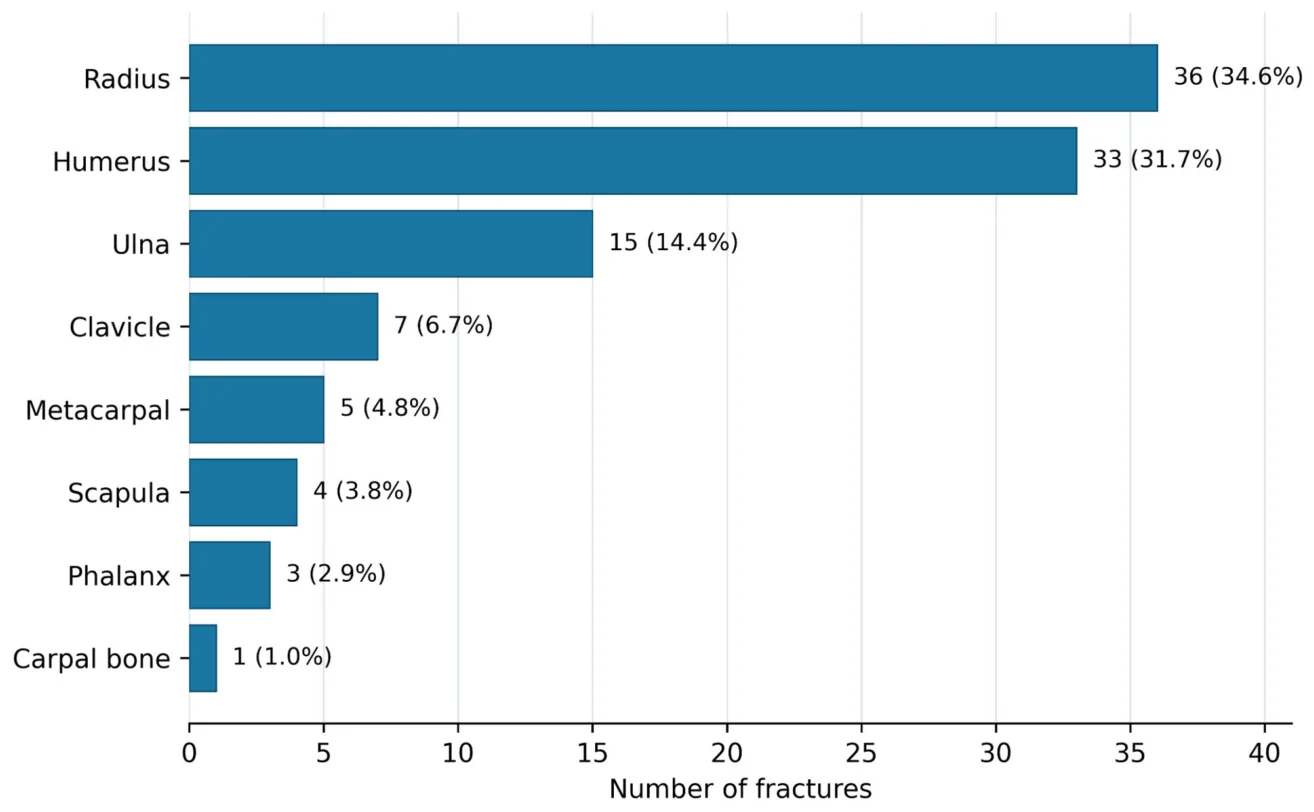
Distribution of upper extremity fractures by affected bone. Percentages were calculated using 104 fractures as the denominator.

A total of 104 upper extremity fractures were identified in 87 patients, corresponding to a mean of 1.20 fractures per patient. Seventeen patients (19.5%) had multiple upper extremity fractures. The radius was the most frequently affected bone (*n* = 36; 34.6%), followed by the humerus *(n* = 33; 31.7%) and ulna (*n* = 15; 14.4%) (Figure 1). Fifty-five fractures (52.9%) were right-sided, and 49 (47.1%) were left-sided.

Eight fractures (7.7%) were open, 74 (71.2%) were displaced, 27 (26.0%) were comminuted, and 19 (18.3%) were intra-articular. Fifty-eight fractures (55.8%) were treated conservatively and 46 (44.2%) surgically (Table 2). Among the 46 surgically treated fractures, plate-and-screw osteosynthesis was used in 41 (89.1%), intramedullary nailing in 3 (6.5%), Kirschner-wire fixation in 1 (2.2%), and isolated screw fixation in 1 (2.2%).

### 3.3. Final Medicolegal Functional Outcomes

The final medicolegal assessment identified no permanent functional impairment in 52 patients (59.8%), permanent functional weakening in 30 (34.5%), and permanent loss of function in 5 (5.7%). Overall, 35 patients had permanent functional impairment (40.2%; 95% CI, 30.6–50.7). Among these 35 patients, restricted range of motion was recorded in 32 (91.4%) and nerve injury in 3 (8.6%). For these 35 patients, the median interval from injury to the definitive medicolegal report was 667 days (IQR, 598.5–737.5 days).

### 3.4. Characteristics Associated with Permanent Functional Impairment

Permanent functional impairment was more frequent among patients with multiple upper extremity fractures than among those with a single fracture (13/17 [76.5%] vs. 22/70 [31.4%]; *p* < 0.001). The impairment rate was 51.7% among patients with at least one displaced fracture and 17.2% among those without a recorded displaced fracture (*p* = 0.002). Permanent impairment occurred in 63.2% of patients with at least one intra-articular fracture and 33.8% of those without an intra-articular fracture (*p* = 0.021). It was also more frequent in the presence of a comminuted fracture (60.0% vs. 34.3%; *p* = 0.040) or an open fracture (83.3% vs. 37.0%; *p* = 0.037).

No statistically significant association between age or sex and permanent functional impairment was detected in this sample. Impairment was more frequent among patients who received surgical or combined treatment, were admitted to hospital, revisited within 30 days, developed a complication, or sustained a life-threatening injury (Table 3). These variables were not entered into the multivariable model because they occurred during the post-injury clinical course.

### 3.5. Multivariable Analysis

**Figure 2:**
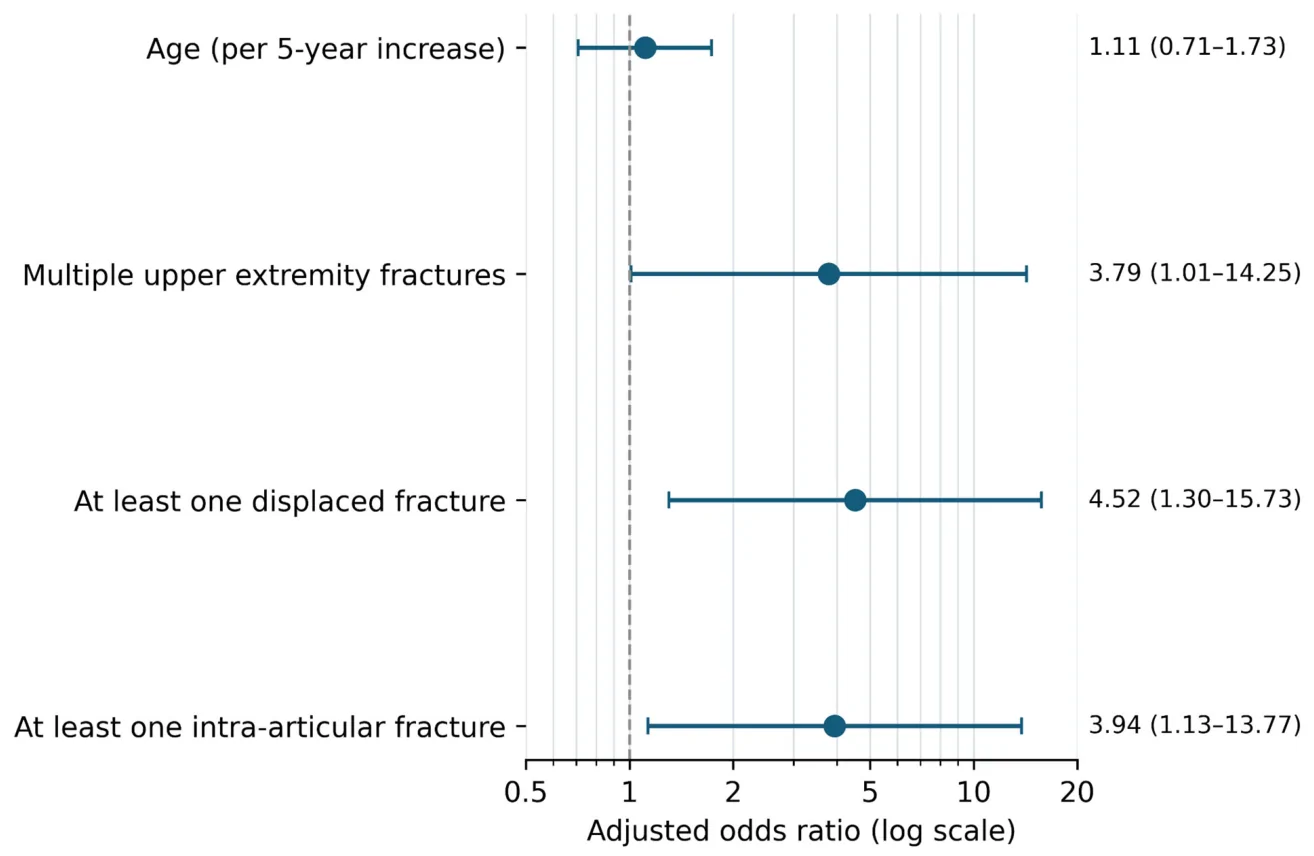
Adjusted odds ratios for variables associated with permanent functional impairment. Points indicate aORs, and horizontal lines indicate 95% CIs. The vertical dashed line indicates the null value. Age was modeled per 5 year increase.

In the logistic regression model that jointly included age, multiple fractures, displacement, and intra-articular extension, multiple upper extremity fractures showed an adjusted association with permanent functional impairment (aOR, 3.79; 95% CI, 1.01–14.25; *p* = 0.049). The aOR was 4.52 (95% CI, 1.30–15.73; *p* = 0.018) for at least one displaced fracture and 3.94 (95% CI, 1.13–13.77; *p* = 0.032) for at least one intra-articular fracture. Each 5 year increase in age did not show a statistically significant adjusted association with the outcome (aOR, 1.11; 95% CI, 0.71–1.73; *p* = 0.661) (Table 4 and Figure 2).

## 4. Discussion

This study evaluated long-term medicolegal functional outcomes together with fracture morphology in patients aged 65 years or older with upper extremity fractures. Permanent functional impairment was identified in 40.2% of patients, with restricted range of motion accounting for most cases. Multiple upper extremity fractures, at least one displaced fracture, and at least one intra-articular fracture remained associated with permanent functional impairment after adjustment for the other variables included in the model, whereas no statistically significant association was detected for age. The wide confidence intervals and limited representation of the oldest patients preclude ranking the relative importance of age and fracture morphology.

The demographic and injury profiles of this cohort differed from those of the general population with geriatric fractures. In a Turkish nationwide series of more than one million geriatric fractures, 66.9% of patients were women, whereas 70.1% of our cohort were men [5]. Our result is consistent with the 69.2% male proportion reported by Kandiş et al. among medicolegal trauma patients aged ≥65 years and with the male predominance previously reported among medicolegal patients with fractures at our center [11,15]. In a separate study of 379 older adults evaluated at our center for physical abuse between 2004 and 2023, 77.6% of victims were women, 79.7% of incidents occurred in the victim’s home, and all perpetrators were known to the victim [21]. Low-energy falls predominate in general emergency department trauma series [22], and falls were also the most common trauma mechanism in the medicolegal geriatric series reported by Kökdener et al. [14]. In contrast, interpersonal violence ranked first in the present study (51.7%), whereas falls ranked third (11.5%). This difference probably reflects selection of patients who sustained an upper extremity fracture and completed a final medicolegal assessment rather than all geriatric trauma patients. The male and violence-predominant profile should therefore be interpreted as a characteristic of this selected medicolegal cohort and not generalized to geriatric fracture epidemiology.

Radius and humerus fractures together accounted for approximately two-thirds of all fractures. In the nationwide geriatric fracture analysis, the wrist was the most common fracture site after the hip and was particularly prominent among patients aged 65–74 years [5]. Proximal humerus fractures are likewise common osteoporotic fractures in older adults [6]. In population-based studies, however, distal radius and proximal humerus fractures are often associated with female sex and low-energy mechanisms [7,23]. The predominance of men and the substantial proportions of interpersonal violence and road traffic collisions in our cohort may have altered the sex and mechanism distributions expected in general geriatric series. Furthermore, our restriction to upper extremity fractures and presentation of distributions at the bone level limit direct comparisons with studies of whole-body fractures or specific anatomic segments.

The high proportions of displaced (71.2%), comminuted (26.0%), and intra-articular (18.3%) fractures indicate a substantial burden of morphologically important injuries. Because these proportions combine different anatomic regions and are affected by medicolegal referral selection, they should not be compared directly with orthopedic series of a single fracture type. Nevertheless, the persistence of associations between displacement or intra-articular extension and permanent functional impairment in multivariable analysis indicates that radiologic assessment should extend beyond merely establishing fracture presence. The cited contemporary models focus on diagnostic accuracy in radiographic fracture detection and automated classification of upper extremity fractures [9,10]. Our findings suggest that future diagnostic decision-support systems may also derive clinical and medicolegal value from structured reporting of fracture count, displacement, and intra-articular extension—features associated with long-term functional outcomes.

Permanent functional impairment occurred in 40.2% of patients, and restricted motion was recorded in 91.4% of affected patients. This outcome represents persistent functional weakening or loss documented in the final medicolegal report rather than a patient-reported outcome measure. Our rate therefore should not be directly compared with orthopedic studies using instruments such as the Disabilities of the Arm, Shoulder, and Hand (DASH) or QuickDASH [24]. Şenol et al., studying unilateral extremity fractures after road traffic collisions, demonstrated variation in sequela rates across treatment groups and showed that disability estimates for the same case could differ between regulatory frameworks [20]. Conversely, studies of older adults with distal radius fractures have reported limited or inconsistent associations between radiographic characteristics and patient-reported functional outcomes [25,26]. These differences support an integrated medicolegal assessment that considers persistent loss of motion, neurologic injury, and effects on daily living rather than radiographic appearance alone.

The adjusted odds ratios were 3.79 for multiple upper extremity fractures, 4.52 for at least one displaced fracture, and 3.94 for at least one intra-articular fracture. Multiple fractures may indicate involvement of several anatomic structures or periarticular regions and a greater rehabilitation burden. Displacement may reflect the initial structural disruption, whereas intra-articular extension may signal incongruity of the articular surface and a risk of stiffness or restricted range of motion. Open and comminuted fractures were also associated with permanent impairment in univariable comparisons, but the limited number of outcome events precluded their addition to the multivariable model. The included morphologic variables should therefore be interpreted as factors showing adjusted associations, not as causal or deterministic criteria. The wide CIs, particularly for multiple fractures, indicate limited precision and the need for validation in larger samples.

No statistically significant association was detected between each 5-year increase in age and permanent functional impairment after adjustment for the other variables included in the model. This result does not imply that older age is clinically irrelevant. The concentration of 77.0% of the cohort in the 65–74-year-old age group, the small number of very old patients, and restriction to patients with a final medicolegal report may have reduced power to detect an age effect. Age may also act through frailty, baseline functional status, osteoporosis, and comorbidities [27]. Because these domains were not measured comprehensively with standardized instruments, the absence of a statistically significant age association should not be interpreted as evidence that age has no effect or as proof that fracture morphology is more important than age.

Surgery was used for 44.2% of fractures, and plate-and-screw osteosynthesis accounted for 89.1% of surgical procedures. An earlier systematic review of distal radius fractures in older patients found worse radiographic results after conservative treatment but no clinically meaningful difference in functional outcomes [28]. A more recent network meta-analysis suggested that volar locking plates may offer advantages in grip strength and some patient-reported outcomes [8,29]. These differing findings indicate that treatment effects depend on fracture type, patient functional demands, and the outcome selected [30]. Although permanent impairment was more frequent among patients receiving surgical or combined treatment in our study, this association should not be interpreted as harm caused by surgery. Displaced, intra-articular, or multiple fractures are more likely to undergo surgery, making confounding by indication likely. Treatment modality was therefore not entered into the multivariable model.

Hospital admission, a revisit within 30 days, complications, and life-threatening injury were also more frequent among patients with permanent functional impairment. Because these variables arise after injury, they should not be considered independent etiologic factors independent of baseline fracture severity. They nevertheless suggest that patients requiring greater healthcare use early in the clinical course may warrant closer monitoring for long-term functional loss. A 30-day revisit may signal post-discharge pain, immobilization problems, treatment-related complications, or insufficient early functional recovery. Future studies should examine revisit diagnoses and rehabilitation trajectories in greater detail.

Strengths of this study include its 10-year period, fracture-level characterization based on original radiology reports, patient-level analyses, and ascertainment of outcomes from definitive medicolegal reports. Limitations include the retrospective single-center setting and restriction to medicolegal referrals. Seven of the 94 initially identified patients lacked accessible follow-up examination records and were excluded; their unknown outcomes introduce possible selection bias, and the direction of this bias cannot be determined. These factors limit generalizability to all older individuals sustaining upper-extremity fractures. The 18-month interval was not a universal eligibility requirement, because patients with complete recovery could receive an earlier definitive report. Radiologic characteristics were abstracted from routine reports without blinded image reassessment, uniform study-specific morphometric thresholds, or an interobserver reliability assessment; variable radiology-report terminology may have introduced misclassification. Functional impairment was determined through routine clinical medicolegal assessment rather than prospectively standardized functional testing, and the clinicians were not formally blinded to previous imaging reports. Baseline upper-limb function, frailty, cognition, rehabilitation exposure, and pre-existing impairment were not consistently assessed using standardized instruments; fracture localization was also not included in the adjusted model. Thus, residual confounding is possible. With only 35 outcome events, the four-variable model yielded wide confidence intervals and potentially unstable estimates, especially for borderline associations. No additional sensitivity analysis was conducted; the stability of the adjusted estimates could therefore not be evaluated across alternative model specifications. The results represent exploratory associations rather than predictive or causal effects, and the 40.2% outcome proportion should not be generalized beyond this medicolegal cohort. Multiple comparisons were not adjusted for, and independent replication is needed.

## 5. Conclusions

In this single-center medicolegal cohort, upper extremity fractures showed a male- and interpersonal violence-predominant pattern. Permanent functional impairment was recorded in 40.2% of these medicolegal patients, and multiple fractures, displacement, and intra-articular extension were associated with this outcome in an exploratory adjusted analysis. These estimates were imprecise, and the study was not designed to develop or validate a prediction model. Standardized documentation of these features in radiology and orthopedic reports may support clinical decisions regarding long-term functional follow-up and rehabilitation. These associations should not be used as automatic decision criteria in medicolegal evaluation; they should be interpreted together with clinical examination, the healing course, and individual functional loss. Multicenter studies with larger samples are needed to validate the findings.

## Figures and Tables

**Table 1:** Patient-level demographic, trauma, clinical, and medicolegal characteristics.

Variable	Result
Demographic characteristics	
Number of patients	87
Age, years, median (IQR)	70 (68–74)
Male sex	61 (70.1)
Female sex	26 (29.9)
Age 65–74 years	67 (77.0)
Age 75–84 years	16 (18.4)
Age ≥ 85 years	4 (4.6)
Living with spouse or family	68 (78.2)
Living alone at home	18 (20.7)
Living in a nursing/residential care facility	1 (1.1)
Presentation and trauma characteristics	
Interpersonal violence	45 (51.7)
Road traffic collision	24 (27.6)
Fall	10 (11.5)
Occupational accident	5 (5.7)
Entrapment or crush injury	2 (2.3)
Other	1 (1.1)
Low-to-moderate-energy trauma	56 (64.4)
High-energy trauma	31 (35.6)
Suspected abuse or neglect	10 (11.5)
Other associated injury	27 (31.0)
Additional fracture outside the upper extremity	15 (17.2)
Multiple upper extremity fractures	17 (19.5)
Comorbidities	
Osteoporosis	9 (10.3)
Dementia	6 (6.9)
Diabetes mellitus	34 (39.1)
Cardiac disease	30 (34.5)
Anticoagulant use	12 (13.8)
Treatment, clinical course, and medicolegal assessment	
Conservative treatment only	49 (56.3)
Surgical treatment only	32 (36.8)
Combined treatment	6 (6.9)
Hospital admission	23 (26.4)
Length of stay, days, median (IQR), admitted patients only	8 (4–11)
30-day revisit	14 (16.1)
At least one complication	15 (17.2)
Life-threatening injury	7 (8.0)
Time from event to forensic medicine evaluation, days, median (IQR)	12 (5.5–46)

Data are presented as *n* (%) unless otherwise indicated. IQR, interquartile range.

**Table 2:** Fracture-level anatomic, morphologic, and treatment characteristics.

Characteristic	*n* (%)
Anatomic distribution	
Radius	36 (34.6)
Humerus	33 (31.7)
Ulna	15 (14.4)
Clavicle	7 (6.7)
Metacarpal	5 (4.8)
Scapula	4 (3.8)
Phalanx	3 (2.9)
Carpal bone	1 (1.0)
Right side	55 (52.9)
Left side	49 (47.1)
Morphologic characteristics	
Open fracture	8 (7.7)
Displaced fracture	74 (71.2)
Comminuted fracture	27 (26.0)
Intra-articular fracture	19 (18.3)
Fracture-dislocation	8 (7.7)
Vascular injury	2 (1.9)
Nerve injury	5 (4.8)
Treatment	
Conservative	58 (55.8)
Surgical	46 (44.2)

The denominator is 104 fracture records.

**Table 3:** Patient-level comparisons according to permanent functional impairment.

Variable	No Impairment (*n* = 52)	Permanent Impairment (*n* = 35)	*p*-Value
Demographic and trauma characteristics			
Age, years, median (IQR)	70 (68–74)	71 (67.5–74)	0.962
Male sex	37 (71.2)	24 (68.6)	0.796
Age ≥ 75 years	12 (23.1)	8 (22.9)	0.981
Interpersonal violence	30 (57.7)	15 (42.9)	0.175
High-energy trauma	16 (30.8)	15 (42.9)	0.248
Other associated injury	15 (28.8)	12 (34.3)	0.591
Additional fracture outside the upper extremity	8 (15.4)	7 (20.0)	0.576
Fracture characteristics at the patient level			
Multiple upper extremity fractures	4 (7.7)	13 (37.1)	<0.001
At least one open fracture	1 (1.9)	5 (14.3)	0.037
At least one displaced fracture	28 (53.8)	30 (85.7)	0.002
At least one comminuted fracture	8 (15.4)	12 (34.3)	0.040
At least one intra-articular fracture	7 (13.5)	12 (34.3)	0.021
Post-injury clinical course			
Surgical or combined treatment	14 (26.9)	24 (68.6)	<0.001
Hospital admission	9 (17.3)	14 (40.0)	0.019
30-day revisit	5 (9.6)	9 (25.7)	0.045
Complication	4 (7.7)	11 (31.4)	0.004
Life-threatening injury	1 (1.9)	6 (17.1)	0.016

Data are presented as *n* (%) unless otherwise indicated. Continuous variables were compared using the Mann–Whitney U test. Pearson’s chi-square test was used when all expected cell counts were ≥5; otherwise, the two-sided Fisher’s exact test was used. Post-injury clinical-course variables were not entered into the multivariable model because of temporal ordering. IQR, interquartile range.

**Table 4:** Multivariable logistic regression for permanent functional impairment.

Variable	aOR	95% CI	*p*-Value
Age, per 5-year increase	1.11	0.71–1.73	0.661
Multiple upper extremity fractures	3.79	1.01–14.25	0.049
At least one displaced fracture	4.52	1.30–15.73	0.018
At least one intra-articular fracture	3.94	1.13–13.77	0.032

The model included 35 outcome events and four explanatory variables. Age was modeled continuously per 5-year increase. aOR, adjusted odds ratio; CI, confidence interval.

## Data Availability

The data are not publicly available because they contain sensitive health and medicolegal information. De-identified data may be available from the corresponding author upon reasonable request and subject to ethics committee and institutional approval.

## Abbreviations

aOR, adjusted odds ratio; CI, confidence interval; IQR, interquartile range.
